# Expansion of Telehealth Availability for Mental Health Care After State-Level Policy Changes From 2019 to 2022

**DOI:** 10.1001/jamanetworkopen.2023.18045

**Published:** 2023-06-13

**Authors:** Ryan K. McBain, Megan S. Schuler, Nabeel Qureshi, Samantha Matthews, Aaron Kofner, Joshua Breslau, Jonathan H. Cantor

**Affiliations:** 1 Department of Medicine, Harvard Medical School, Boston, Massachusetts; 2 Division of Healthcare Delivery, RAND Corporation, Arlington, Virginia; 3 Division of Healthcare Delivery, RAND Corporation, Santa Monica, California; 4 Division of Healthcare Delivery, RAND Corporation, Pittsburgh, Pennsylvania

## Abstract

**Importance:**

Although telehealth services expanded rapidly during the COVID-19 pandemic, the association between state policies and telehealth availability has been insufficiently characterized.

**Objective:**

To investigate the associations between 4 state policies and telehealth availability at outpatient mental health treatment facilities throughout the US.

**Design, Setting, and Participants:**

This cohort study measured whether mental health treatment facilities offered telehealth services each quarter from April 2019 through September 2022. The sample comprised facilities with outpatient services that were not part of the US Department of Veterans Affairs system. Four state policies were identified from 4 different sources. Data were analyzed in January 2023.

**Exposures:**

For each quarter, implementation of the following policies was indexed by state: (1) payment parity for telehealth services among private insurers; (2) authorization of audio-only telehealth services for Medicaid and Children’s Health Insurance Program (CHIP) beneficiaries; (3) participation in the Interstate Medical Licensure Compact (IMLC), permitting psychiatrists to provide telehealth services across state lines; and (4) participation in the Psychology Interjurisdictional Compact (PSYPACT), permitting clinical psychologists to provide telehealth services across state lines.

**Main Outcome and Measures:**

The primary outcome was the probability of a mental health treatment facility offering telehealth services in each quarter for each study year (2019-2022). Information on the facilities was obtained from the Mental Health and Addiction Treatment Tracking Repository based on the Substance Abuse and Mental Health Services Administration Behavioral Health Treatment Service Locator. Separate multivariable fixed-effects regression models were used to estimate the difference in the probability of offering telehealth services after vs before policy implementation, adjusting for characteristics of the facility and county in which the facility was located.

**Results:**

A total of 12 828 mental health treatment facilities were included. Overall, 88.1% of facilities offered telehealth services in September 2022 compared with 39.4% of facilities in April 2019. All 4 policies were associated with increased odds of telehealth availability: payment parity for telehealth services (adjusted odds ratio [AOR], 1.11; 95% CI, 1.03-1.19), reimbursement for audio-only telehealth services (AOR, 1.73; 95% CI, 1.64-1.81), IMLC participation (AOR, 1.40, 95% CI, 1.24-1.59), and PSYPACT participation (AOR, 1.21, 95% CI, 1.12-1.31). Facilities that accepted Medicaid as a form of payment had lower odds of offering telehealth services (AOR, 0.75; 95% CI, 0.65-0.86) over the study period, as did facilities in counties with a higher proportion (>20%) of Black residents (AOR, 0.58; 95% CI, 0.50-0.68). Facilities in rural counties had higher odds of offering telehealth services (AOR, 1.67; 95% CI, 1.48-1.88).

**Conclusion and Relevance:**

Results of this study suggest that 4 state policies that were introduced during the COVID-19 pandemic were associated with marked expansion of telehealth availability for mental health care at mental health treatment facilities throughout the US. Despite these policies, telehealth services were less likely to be offered in counties with a greater proportion of Black residents and in facilities that accepted Medicaid and CHIP.

## Key Points

**Question:**

Were state-level policy changes from 2019 to 2022 associated with the expansion of telehealth services at mental health treatment facilities throughout the US?

**Findings:**

In this cohort study of 12 828 mental health treatment facilities, 4 state policies pertaining to payment parity, audio-only telehealth service reimbursement, and interstate licensure compacts were associated with increased telehealth availability during the COVID-19 pandemic in the US. However, access disparities persisted in counties with a higher proportion of Black residents and among Medicaid and Children's Health Insurance Program recipients.

**Meaning:**

Findings of this study suggest that although changes to state policies may facilitate greater access to mental health treatment via telehealth, disparities in access persist.

## Teaser

This cohort study evaluates telehealth use and accessibility at US mental health treatment facilities before and during the COVID-19 pandemic.

## Introduction

Telehealth, or the provision of remote medical care and education by means of telecommunications,^1^ expanded rapidly throughout the COVID-19 pandemic, particularly for outpatient psychiatric services. Among individuals with employer-based insurance, there was a 20-fold increase in telehealth service use for mental health needs.^2^ In a recent national survey administered by the American Medical Association, more than two-thirds (69%) of contacted medical practices stated that they intended to sustain telehealth as a permanent fixture of their service portfolio, with more than half of physicians reporting they would continue using telehealth services.^3^ The benefits of telehealth include increased flexibility and convenience for patients^4,5,6,7^ and improved access to specialty care.^8,9^ During the COVID-19 pandemic, telehealth also bolstered the supply of services when in-person care was restricted.^10,11^

While the COVID-19 pandemic served as a catalyst for the expansion of telehealth, state legislation and executive orders may have made the transition to telehealth economically and logistically feasible. For example, Medicaid agencies in 33 states authorized reimbursement for audio-only telehealth services among Medicaid and Children’s Health Insurance Program (CHIP) beneficiaries.^12^ In addition, executive orders established payment parity, the requirement that health plans provide equal reimbursement for telehealth and in-person services.^13^ Similarly, many states joined compacts, such as the Interstate Medical Licensure Compact (IMLC) and the Psychology Interjurisdictional Compact (PSYPACT), which allow psychiatrists and clinical psychologists, respectively, to practice telehealth across state lines.^14,15^

The extent and timing of policy adoption varied widely across states.^16,17^ This temporal and geographic heterogeneity provides an opportunity to assess the associations between major state policies and expansion of telehealth availability throughout the US. Knowing whether and to what extent these policies were factors in expansion of telehealth services for mental health treatment is important not only for judging their merit but also for understanding the potential ramifications of ending these policies, which may occur at the expiration of the national COVID-19 public health emergency (May 11, 2023).^18^ This knowledge is particularly relevant in the context of telehealth expansion, as many states used temporary measures that are set to expire.

To our knowledge, this study was the first to investigate the associations between 4 state policies and telehealth availability at outpatient mental health treatment facilities throughout the US. To this end, we examined the timing between when state policies were enacted and when mental health facilities began offering telehealth services.

## Methods

Facility-based data were used to assess expansion of telehealth availability for mental health care during the COVID-19 pandemic. The RAND Human Subjects Protection Committee deemed this cohort study exempt from review, as it did not involve human participants. We followed the Strengthening the Reporting of Observational Studies in Epidemiology (STROBE) reporting guideline.

### Study Population

We gathered information on outpatient mental health treatment facilities throughout the US from the Mental Health and Addiction Treatment Tracking Repository (MATTR). This repository provides longitudinal data on characteristics of mental health treatment facilities based on the Substance Abuse and Mental Health Services Administration (SAMHSA) Behavioral Health Treatment Service Locator.^19^ The literature estimates that the Service Locator file contains most of the mental health treatment facilities in the US.^20^ For the purposes of this study, we restricted the sample to those facilities with outpatient services that were not part of the US Department of Veterans Affairs system. The final analytic sample contained 12 828 mental health treatment facilities.

### Exposures

We identified 4 state policies for inclusion, each of which is theorized to have an association with shaping clinicians’ decisions to offer telehealth services. eAppendixes 1 to 4 in Supplement 1 contain documentation for each policy within each US state.

One policy pertained to whether a state required, through executive order or legislation, payment parity for telehealth services among private insurers. We hypothesized that mental health treatment facilities would be more inclined to offer telehealth services if reimbursement levels were commensurate with those for in-person services. The dates when the state policies were ratified and the contents of individual policies were identified and cross-referenced from 4 sources: Manatt,^21^ the American Psychological Association,^22^ the Center for Connected Health Policy,^23,24^ and individual state legislative websites.

Two policies involved 2 interstate compacts aimed at liberalizing telehealth availability across state lines: PSYPACT and IMLC, which permit eligible clinical psychologists and physicians (including psychiatrists), respectively, to practice telehealth across state lines. We hypothesized that state participation in these compacts would increase the availability of telehealth services at mental health treatment facilities as these compacts allow participating clinicians to reach a wider patient population. We identified the originating date of ratified interstate compacts from the PSYPACT^14^ and IMLC^25^ websites.

Another policy included reimbursement authorization for audio-only telehealth services among Medicaid and CHIP beneficiaries.^26^ This policy decision rested with individual state Medicaid agencies, whose websites we reviewed. We hypothesized that mental health treatment facilities would be more inclined to offer telehealth services if they could reach Medicaid and CHIP beneficiaries who had no access to smartphones or reliable broadband internet.

### Outcome

The primary outcome was the probability of a mental health treatment facility offering telehealth services in each quarter of each year from April 2019 to September 2022. This information was tracked using MATTR, which catalogs the dates and responses from facilities to SAMHSA regarding telehealth’s availability as a service modality. We selected quarterly intervals because facilities are not obligated to report changes in service delivery to SAMHSA on a daily or weekly basis; rather, they are expected to provide routine updates. After reviewing SAMHSA data files, we determined that quarterly intervals were commensurate with the temporal resolution of reporting. Data were unavailable for the third quarter of 2021.

### Covariates

To account for variation in mental health treatment facility characteristics, we included 2 indicator variables: one indicating whether the facility accepted Medicaid as a form of payment, and the other indicating whether the facility self-identified as a community mental health center (CMHC).^27^ Using data from the 2020 American Community Survey,^28^ we also incorporated characteristics from counties in which each facility was located. The data included the percentage of residents who self-identified as Black individuals (4 groups: ≤5, >5-10, >10-20, or >20), percentage of residents who self-identified as Hispanic individuals (4 groups: ≤5, >5-10, >10-20, or >20), percentage of residents aged 0 to 17 years (dichotomized: ≤25 or >25), percentage of residents 65 years or older (dichotomized: ≤15 or >15), percentage of households without internet access (dichotomized: ≤15 or >15), and percentage of residents participating in public assistance programs, such as the Supplemental Nutrition Assistance Program (dichotomized: ≤10 or >10). County urbanicity was also classified based on the Rural-Urban Continuum Code (RUCC), with counties classified as urban (RUCC 1-3) or rural (RUCC 4-9). Black race and Hispanic ethnicity were selected as covariates because they represented the 2 largest population groups among whom disparities in access to health services are consistently documented.

We included an indicator for state Medicaid expansion status.^29^ Based on the Kaiser Family Foundation database of state Medicaid expansion dates,^29^ we created quarterly indicators denoting whether a state had enacted Medicaid expansion.

### Statistical Analysis

We used longitudinal, multivariable fixed-effects logistic regression to estimate the difference in the probability that mental health treatment facilities would offer telehealth services in quarters after policy implementation compared with quarters before policy implementation.^30^ Separate regression models were estimated for each state policy. In addition to the dichotomous policy indicator (yes or no), we included all facility-, county-, and state-level covariates. Fixed effects were included for each state and year, and cluster-robust SEs were clustered at the facility level.

In addition to the main fixed-effects models, we conducted 2 secondary analyses examining effect modification. First, in a single model with no policy indicators, we initiated interaction between 3 county-level characteristics (rural vs urban, percentage of Black residents, and percentage of Hispanic residents) and year to assess whether temporal trends in telehealth availability over the analytic period differed according to the counties’ racial and ethnic composition and urbanicity. Second, considering each policy separately, we generated interaction between each of these 3 county-level characteristics and the policy indicator to assess whether the associations between policies and expansion of telehealth availability differed by the racial and ethnic composition and urbanicity of the counties.

Significance testing for interaction terms was conducted using omnibus χ^2^ tests, and we estimated predictive margins of telehealth availability that corresponded to subgroups defined by these interaction terms. A 2-sided *P* < .05 indicated statistical significance. All analyses were conducted in January 2023 using Stata, version 17.1 (StataCorp LLC).^31^

## Results

### Mental Health Treatment Facility Characteristics 

Over the study period, the percentage of mental health treatment facilities offering telehealth services more than doubled from 39.4% in 2019 (quarter 2) to 88.1% in 2022 (quarter 3). Of the 12 828 mental health treatment facilities included, 88.2% accepted Medicaid and 26.3% operated as a CMHC. A preponderance of mental health treatment facilities were in urban counties (75.0%) and counties in which 10% or less of the population were Black (61.9%) and Hispanic (56.3%) residents. Additional information on facility characteristics is provided in Table 1.

**Table 1.** Mental Health Treatment Facility Characteristics

Characteristic	All facilities, No. (%)^a^	Facilities offering telehealth, No. (%)^a^
Facility-level characteristics		
Total No.	12 828 (100)	10 284 (100)
Accepted Medicaid	11 311 (88.2)	9126 (88.7)
CMHC	3373 (26.3)	2930 (28.5)
County-level characteristics		
Total No.	12 828 (100)	10 284 (100)
Urbanicity		
Urban	9623 (75.0)	7492 (72.9)
Rural	3205 (25.0)	2792 (27.2)
% of Black residents^b^		
≤5	5518 (43.0)	4594 (44.7)
>5-10	2430 (18.9)	1944 (18.9)
>10-20	2259 (17.6)	1745 (17.0)
>20	2621 (20.4)	2001 (19.5)
% of Hispanic residents^b^		
≤5	4103 (32.0)	3380 (32.9)
>5-10	3115 (24.3)	2491 (24.2)
>10-20	2579 (20.1)	2038 (19.8)
>20	3031 (23.6)	2375 (23.1)
% of Residents aged ≥65 y		
≤15	4476 (34.9)	3576 (34.8)
>15	8352 (65.1)	6708 (65.2)
% of Residents aged 0-17 y		
≤25	11 130 (86.8)	8870 (86.3)
>25	1698 (13.2)	1414 (13.8)
% of Residents participating in public assistance programs		
≤10	4347 (33.9)	3507 (34.1)
>10	8481 (66.1)	6777 (65.9)
% of Households without internet access		
≤15%	9083 (70.8)	7129 (69.3)
>15%	3745 (29.2)	3155 (30.7)

Abbreviation: CMHC, community mental health center.

^a^ Based on responses from the end of the analytic period: July to September 2022.

^b^ Black race and Hispanic ethnicity were self-identified by participants in the 2020 American Community Survey.^28^

**Figure 1.**
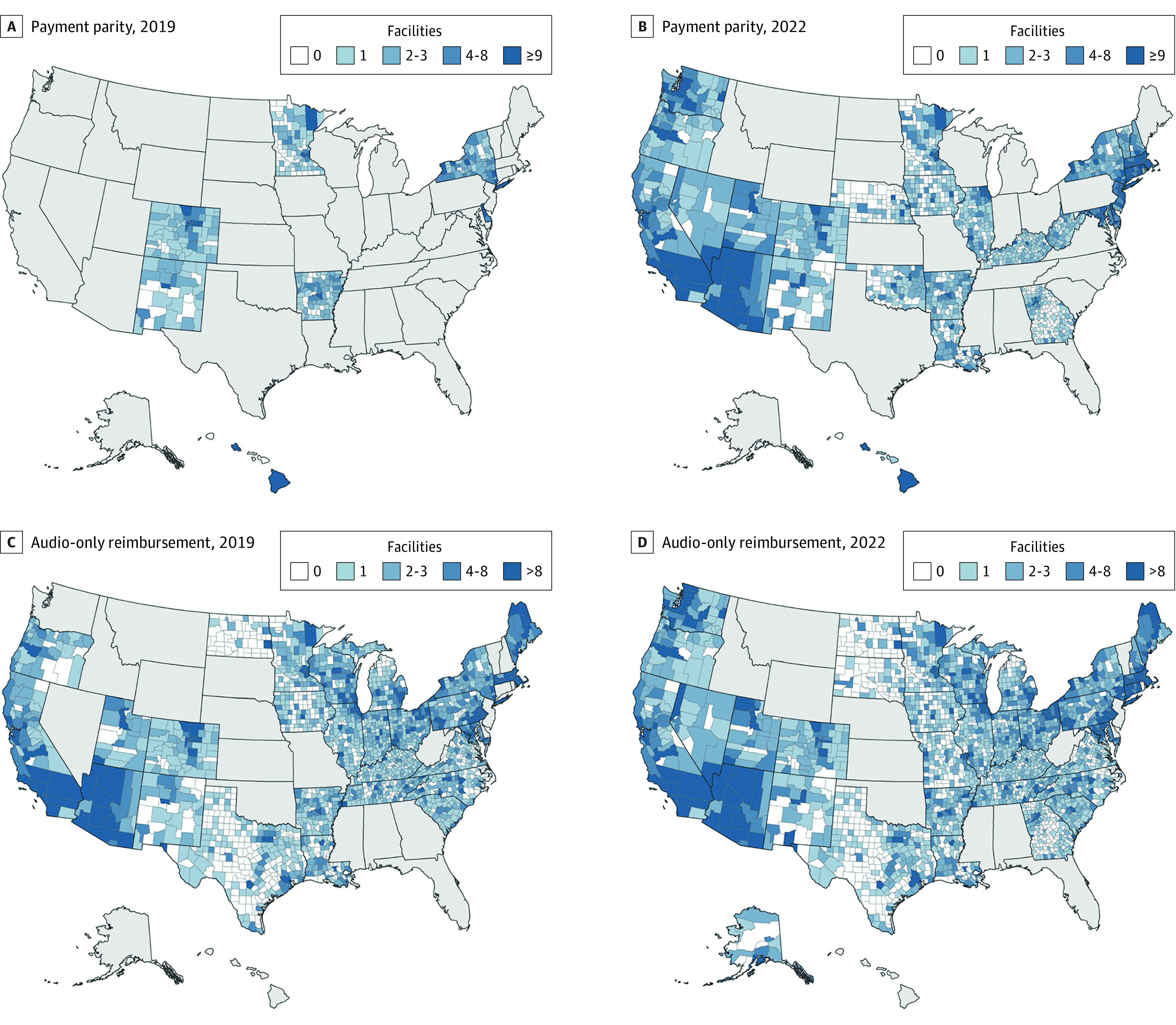
Adoption of Telehealth Payment Parity and Audio-Only Telehealth Services From 2019 to 2022

**Figure 2.**
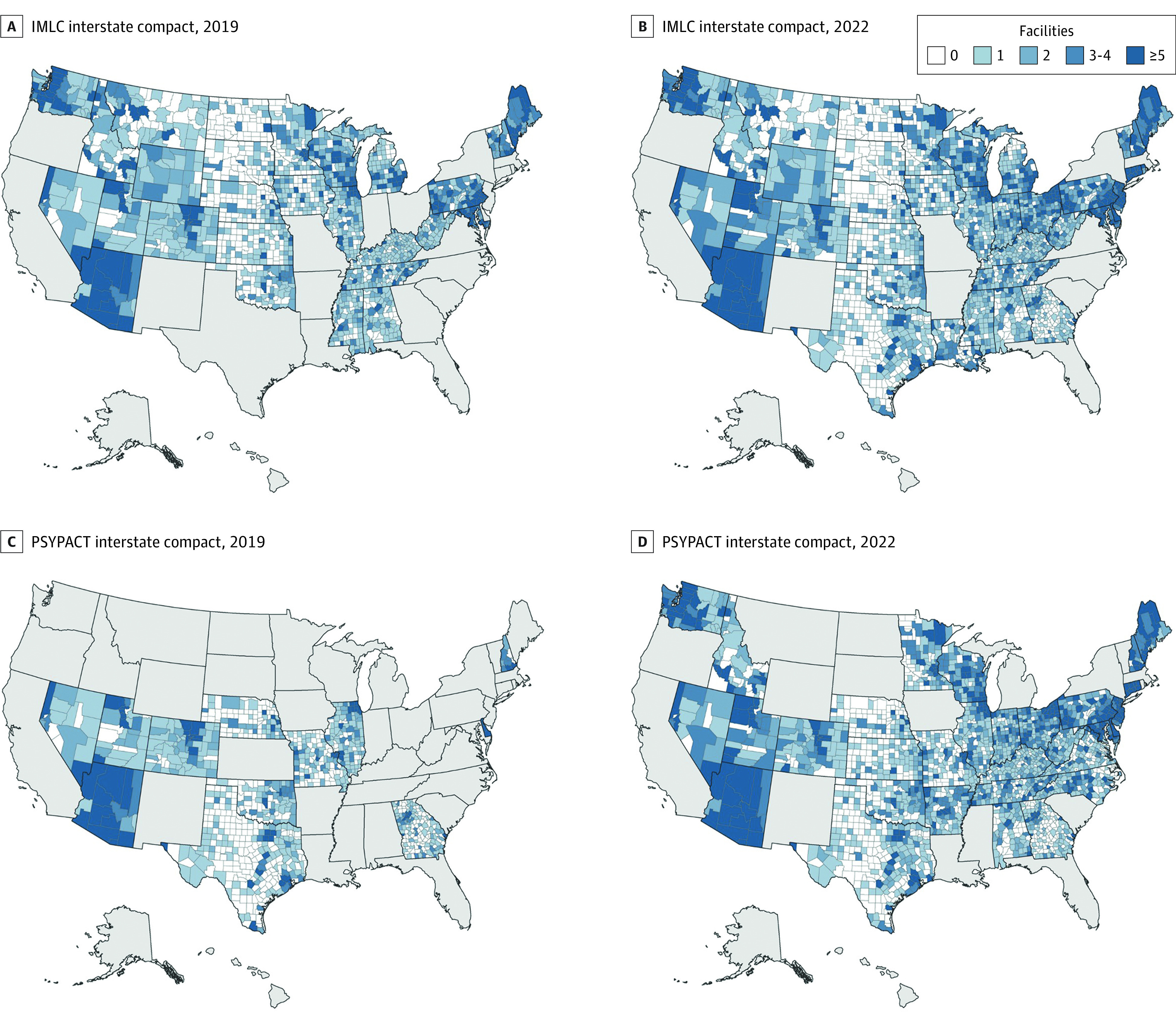
Adoption of Interstate Compacts for Telehealth Services From 2019 to 2022 IMLC indicates Interstate Medical Licensure Compact; PSYPACT, Psychology Interjurisdictional Compact.

Of the 50 states and Washington, DC, the number of states with payment parity policies for telehealth services increased from 6 (11.8%) to 28 (54.9%), and the number of states with audio-only authorization increased from 0 (0%) to 33 (64.7%). Participation in IMLC increased from 28 (54.9%) to 38 (74.5%) states, and participation in PSYPACT increased from 7 (13.7%) to 32 (62.7%) states. Figure 1 and Figure 2 show the density of mental health treatment facilities in the US.

### Policy Associations With Telehealth Availability

As shown in Table 2, all 4 state policies were associated with significantly increased odds of a mental health treatment facility offering telehealth services based on multivariable regression analyses adjusted for facility-level and county-level characteristics. Facilities in states that required payment parity for telehealth (vs in-person) services had 11% higher odds of offering telehealth services (adjusted odds ratio [AOR], 1.11; 95% CI, 1.03-1.19). Facilities in states that allowed reimbursement for audio-only telehealth visits had 73% higher odds of offering telehealth services (AOR, 1.73; 95% CI, 1.64-1.81). Availability of telehealth services at mental health treatment facilities was associated with state participation in both IMLC (AOR, 1.40; 95% CI, 1.24-1.59) and PSYPACT (AOR, 1.21; 95% CI, 1.12-1.31).

**Table 2.** Adjusted Odds of Telehealth Availability at Mental Health Treatment Facilities^a^

Measure	Payment parity		Audio-only authorization		IMLC		PSYPACT	
	AOR (95% CI)	*P* value	AOR (95% CI)	*P* value	AOR (95% CI)	*P* value	AOR (95% CI)	*P* value
State level								
Policy ratification	1.11 (1.03-1.19)	<.001	1.73 (1.64-1.81)	<.001	1.40 (1.24-1.59)	<.001	1.21 (1.12-1.31)	<.001
Medicaid expansion	0.66 (0.37-1.20)	.17	0.67 (0.38-1.19)	.17	0.67 (0.37-1.21)	.19	0.68 (0.37-1.23)	.20
County-level characteristics								
Rural^b^	1.67 (1.48-1.88)	<.001	1.68 (1.49-1.89)	<.001	1.67 (1.48-1.88)	<.001	1.67 (1.48-1.88)	<.001
% of Black residents								
≤5	1 [Reference]	NA	1 [Reference]	NA	1 [Reference]	NA	1 [Reference]	NA
>5-10	0.87 (0.78-0.97)	.01	0.87 (0.77-0.97)	.01	0.87 (0.78-0.97)	.01	0.87 (0.78-0.97)	.01
>10-20	0.78 (0.69-0.89)	<.001	0.78 (0.68-0.80)	<.001	0.78 (0.69-0.89)	<.001	0.78 (0.69-0.89)	<.001
>20	0.58 (0.50-0.68)	<.001	0.58 (0.50-0.67)	<.001	0.58 (0.50-0.68)	<.001	0.58 (0.50-0.68)	<.001
% of Hispanic residents								
≤5	1 [Reference]	NA	1 [Reference]	NA	1 [Reference]	NA	1 [Reference]	NA
>5-10	0.92 (0.82-1.03)	.14	0.92 (0.82-1.03)	.14	0.92 (0.82-1.03)	.14	0.92 (0.82-1.03)	.14
>10-20	0.91 (0.79-1.04)	.18	0.91 (0.79-1.04)	.17	0.91 (0.79-1.04)	.18	0.91 (0.79-1.04)	.18
>20	0.86 (0.73-1.01)	.07	0.86 (0.73-1.01)	.06	0.86 (0.73-1.01)	.07	0.86 (0.73-1.01)	.07
% of Residents aged 0-17 y								
≤25	1 [Reference]	NA	1 [Reference]	NA	1 [Reference]	NA	1 [Reference]	NA
>25	1.16 (1.00-1.34)	.05	1.16 (1.00-1.35)	.05	1.16 (1.00-1.34)	.05	1.16 (1.00-1.34)	.05
% of Residents aged ≥65 y								
≤15	1 [Reference]	NA	1 [Reference]	NA	1 [Reference]	NA	1 [Reference]	NA
>15	0.90 (0.83-1.02)	.10	0.90 (0.83-1.02)	.10	0.92 (0.83-1.02)	.10	0.92 (0.83-1.02)	.10
% of Residents participating in public assistance programs								
≤10	1 [Reference]	NA	1 [Reference]	NA	1 [Reference]	NA	1 [Reference]	NA
>10	1.11 (1.01-1.21)	.03	1.11 (1.01-1.22)	.03	1.11 (1.01-1.21)	.03	1.11 (1.01-1.21)	.03
% of Households without internet access								
≤15	1 [Reference]	NA	1 [Reference]	NA	1 [Reference]	NA	1 [Reference]	NA
>15	1.16 (1.05-1.30)	.01	1.17 (1.05-1.30)	.01	1.17 (1.05-1.30)	.01	1.17 (1.05-1.30)	.01
Facility-level characteristics								
Accepted Medicaid	0.75 (0.65-0.86)	<.001	0.75 (0.65-0.86)	<.001	0.75 (0.65-0.86)	<.001	0.75 (0.65-0.86)	<.001
CMHC	1.40 (1.28-1.53)	<.001	1.40 (1.27-1.53)	<.001	1.40 (1.28-1.53)	<.001	1.40 (1.28-1.53)	<.001

Abbreviations: AOR, adjusted odds ratio; CMHC, community mental health center; IMLC, Interstate Medical Licensure Compact; NA, not applicable; PSYPACT, Psychology Interjurisdictional Compact.

^a^ All regression models included state and year fixed effects. The SEs were clustered at the facility level.

^b^ Reference group: urban.

Across all 4 regression models, facility-level and county-level characteristics exhibited consistent associations with telehealth availability. Specifically, mental health treatment facilities that accepted Medicaid had significantly lower odds of offering telehealth services compared with facilities that did not accept Medicaid (AOR, 0.75; 95% CI, 0.65-0.86), and CMHCs were significantly more likely to offer telehealth services than other facility types (AOR, 1.40; 95% CI, 1.28-1.53). Facilities in rural counties were significantly more likely to offer telehealth services compared with facilities in urban counties (AOR, 1.67; 95% CI, 1.48-1.88). Facilities in counties in which more than 10% of residents were participating in public assistance programs were significantly more likely to offer telehealth services (AOR, 1.11; 95% CI, 1.01-1.21), as were facilities in counties in which more than 15% of households lacked internet access (AOR, 1.16; 95% CI, 1.05-1.30).

The percentage of Black residents in the county was inversely associated with telehealth availability. Compared with counties with 5% or fewer Black residents, counties with more than 20% Black residents had 42% lower odds of offering telehealth services (AOR, 0.58; 95% CI, 0.50-0.68). In contrast, the proportions of county residents who identified as Hispanic individuals, were aged 0 to 17 years, or were 65 years or older were not associated with the odds of a facility offering telehealth services.

### Effect Modification

**Figure 3.**
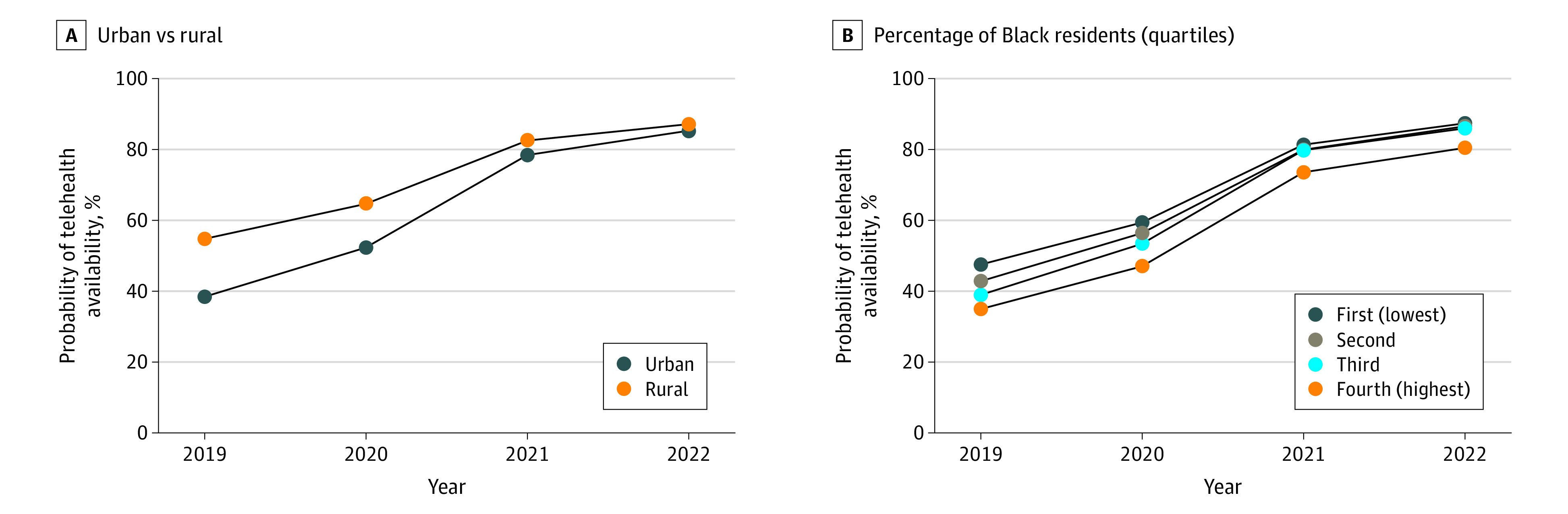
Telehealth Policy Adoption by County Characteristics From 2019 to 2022

In the first set of interaction models between county-level characteristics (rural vs urban, percentage of Black residents, and percentage of Hispanic residents) and year, we found significant evidence of differential time trends by rurality and percentage of Black residents. As shown in Figure 3, the rural-urban gap in telehealth availability was pronounced in 2019 (55% of rural facilities vs 38% of urban facilities) but systematically narrowed over the study period (2022: 88% of rural facilities vs 85% of urban facilities). Additionally, differences in telehealth availability across counties based on the percentage of Black residents decreased over the study period such that availability was similar in 2022 for counties with 20% or fewer Black residents. However, throughout the study period, counties with more than 20% Black residents had consistently lower telehealth availability.

In the second set of interaction models between policies of interest and county-level characteristics, we found that for 3 of 4 policies (payment parity, audio-only authorization, and IMLC participation), policy implementation was associated with greater expansion of telehealth availability in urban vs rural counties (eAppendix 4 in Supplement 1). Similarly, payment parity and audio-only authorization policies were associated with greater expansion of telehealth availability in counties with a higher proportion (>20%) of Black residents. Audio-only authorization was also associated with greater expansion of telehealth availability in communities with a higher proportion (>20%) of Hispanic residents.

## Discussion

To our knowledge, this study was one of the first investigations into the association between state policies and expanded telehealth availability during the COVID-19 pandemic. We found that all 4 policies were associated with greater telehealth availability over time.

Medicaid and CHIP reimbursement for audio-only telehealth services was associated with a 73% higher likelihood of telehealth availability. This result is notable for 2 reasons. First, apart from Medicare, Medicaid is the largest insurer in the US, with more than 90 million enrollees as of 2021.^32^ Second, Medicaid serves a high-needs population: approximately 50% of Medicaid beneficiaries who are eligible based on a disability have 1 or more behavioral health diagnoses, and Medicaid is the largest single payer for behavioral health services.^33,34^ However, we also found that mental health treatment facilities that accepted Medicaid as a form of insurance were, overall, less likely to offer telehealth services throughout the analytic period regardless of the status of any given policy. This finding is consistent with prior studies indicating that Medicaid and CHIP recipients may have reduced access to outpatient care compared with individuals with private insurance.^35^

State participation in PSYPACT and IMLC corresponded to 21% (AOR, 1.21) and 40% (AOR, 1.40) greater likelihood, respectively, of a facility expanding to offer telehealth services, whereas payment parity was associated with an 11% (AOR, 1.11) greater likelihood. Interstate compacts offer a vehicle for facilities and their clinicians to broaden reach of services, although the extent to which this expansion occurs has not been well documented. However, it is likely that these associations are most consistently observed in urban hubs close to state boundaries, such as New York City or Washington, DC.^36^ Clinicians may also preferentially select clients from other states if they pay out of pocket or present with a more generous insurer.^37^

We anticipated that payment parity would be associated with expanded telehealth availability, which was not observed. One explanation for this finding is that, historically, enforcement of parity laws has been weak,^38^ and mental health treatment facilities may have been skeptical about the extent to which private insurers would be forthcoming with telehealth reimbursement. Another explanation is that even prior to the payment parity policy, insurers may have offered partial reimbursement. Payment parity remains contentious, with some arguing that telehealth may be lower in cost and lower in value than in-person care, and reimbursement rates should reflect this.^13^ Payment parity is also the policy that is most likely to regress as it tends to rely on temporary executive orders.^16^

This study found some evidence that state policies were differentially associated with expansion of telehealth availability in some demographic groups compared with others. For example, audio-only authorization was associated with larger expansion of telehealth availability in counties with more than 20% Black residents than in counties with a predominantly White population (≤5% Black residents). However, time trends also showed that, over the analytic period, disparities in telehealth availability persisted for several groups, including Medicaid and CHIP beneficiaries and Black residents. Facilities that accepted Medicaid and CHIP were approximately 25% less likely to offer telehealth services, and facilities in counties with a high proportion (>20%) of Black residents were 42% less likely to offer telehealth services compared with facilities in counties with predominantly White residents (≤5% Black residents). Taken together, these findings indicated that state policies were associated with increased telehealth coverage; however, targeted policies are still required to advance equity in access among underserved populations.

Additionally, we found evidence that greater telehealth availability in rural vs urban counties shrank over the study period. Consistent with prior research,^39^ the present study showed that before the pandemic, telehealth was a modality that was most common in rural areas. However, throughout the pandemic, telehealth availability increased more rapidly in urban areas. In a post-COVID-19 era, this availability raises questions about equity in access, as those living in rural areas may once again have greater need for reliance on telehealth services.

Overall, the observed rapid growth in telehealth services for mental health care, from 39.4% in 2019 to 88.1% in 2022, suggests that state policies served an important role. This role should be viewed within the broader context of increased acceptability of telehealth over the same period^40^ as well as accumulating evidence that telehealth is an effective modality for delivering many specific mental health services.^41^

### Limitations

This study has several limitations. First, information on availability of telehealth services relied on mental health treatment facilities reporting to SAMHSA. Second, when measuring the association between policies and telehealth availability, we used the date on which the policy was formalized or ratified. However, the policies may take weeks or months to implement. Similarly, the contents of individual policies varied across states and over time. For this analysis, we simplified policies into discrete categories. Third, omitted variable bias could alter the estimation effects. We attempted to address this issue by incorporating facility-level and county-level characteristics. Fourth, the analysis focused on telehealth availability for outpatient services; it did not provide insights on service volume or the associations with inpatient services.

## Conclusions

In this national cohort study, 4 state policies were associated with a consistently greater likelihood of telehealth availability at mental health treatment facilities in the US. Despite these policies, certain groups remained underserved: Medicaid and CHIP beneficiaries and Black individuals. This finding underscores the need for targeted local legislation that could sustain and further expand access to telehealth services.

## Notes

Supplement 1. **eAppendix 1.** Telehealth State Policy Start Dates **eAppendix 2.** Assessing Differential Time Trends in Telehealth Availability by Rurality and Race/Ethnicity **eAppendix 3.** Assessing Differential Associations Between State Policy and Telehealth Availability by Rurality and Race/Ethnicity **eAppendix 4.** Predictive Margins Assessing Differential Associations Between State Policy and Telehealth Availability by Rurality and Race/Ethnicity

Supplement 2.
Data Sharing Statement
