# Supplementary material for: Expansion of Telehealth Availability for Mental Health Care After State-Level Policy Changes From 2019 to 2022
Source: JAMA Netw Open. 2023 Jun 13;6(6):e2318045. doi: 10.1001/jamanetworkopen.2023.18045 (PMC10265313; doi:10.1001/jamanetworkopen.2023.18045)
Supplement: Supplement 1. — eAppendix 1. Telehealth State Policy Start Dates eAppendix 2. Assessing Differential Time Trends in Telehealth Availability by Rurality and Race/Ethnicity eAppendix 3. Assessing Differential Associations Between State Policy and Telehealth Availability by Rurality and Race/Ethnicity eAppendix 4. Predictive Margins Assessing Differential Associations Between State Policy and Telehealth Availability by Rurality and Race/Ethnicity [file jamanetwopen-e2318045-s001.pdf]

Supplemental Online Content

McBain RK, Schuler MS, Qureshi N, et al. Expansion of telehealth availability for mental health care after state-level policy changes from 2019 to 2022. *JAMA Netw Open*. 2023;6(6):e2318045. doi:10.1001/jamanetworkopen.2023.18045

eAppendix 1. Telehealth State Policy Start Dates

eAppendix 2. Assessing Differential Time Trends in Telehealth Availability by Rurality and Race/Ethnicity

eAppendix 3. Assessing Differential Associations Between State Policy and Telehealth Availability by Rurality and Race/Ethnicity

eAppendix 4. Predictive Margins Assessing Differential Associations Between State Policy and Telehealth Availability by Rurality and Race/Ethnicity

This supplemental material has been provided by the authors to give readers additional information about their work.

eAppendix 1. Telehealth State Policy Start Dates

US State	*Payment Parity*	*Audio-Only Authorization*	*IMLC[a] Compact*	*PSYPACT[b] Compact*
Alabama	na[c]	na	5/19/15	3/18/21
Alaska	na	7/14/22	na	na
Arizona	3/25/20	3/25/20	5/11/16	5/17/16
Arkansas	1/1/18	3/27/20	na	4/25/21
California	3/1/20	4/30/20	na	na
Colorado	1/1/17	4/30/20	6/8/16	4/12/18
Connecticut	6/8/21	6/8/21	5/13/22	5/24/22
Delaware	7/7/15	na	6/23/21	6/27/19
District of Columbia	na	3/19/20	3/7/17	3/16/21
Florida	na	na	na	na
Georgia	1/1/20	5/4/2021	9/16/21	4/23/19
Hawaii	12/31/16	na	na	na
Idaho	na	na	3/25/15	3/23/22
Illinois	3/19/20	3/19/20	7/21/15	8/22/18
Indiana	na	3/6/20	3/10/22	3/10/22
Iowa	1/1/21	9/18/20	7/2/15	na
Kansas	na	na	5/13/16	5/17/21
Kentucky	1/1/22	3/11/20	12/13/18	3/18/21
Louisiana	1/1/21	3/17/20	7/1/21	na
Maine	na	3/16/20	4/6/17	6/22/21
Maryland	7/1/21	4/3/20	1/19/18	5/18/21
Massachusetts	1/1/21	4/1/20	na	na
Michigan	na	3/20/20	6/1/19	na
Minnesota	1/1/17	12/14/20	5/19/15	5/25/21
Mississippi	na	na	5/17/16	na
Missouri	na	3/8/22	na	6/1/18
Montana	na	na	4/8/15	na
Nebraska	4/21/21	na	1/5/17	4/23/18
Nevada	10/1/21	6/4/21	5/27/15	5/26/17
New Hampshire	7/21/20	7/21/22	5/5/16	7/10/19
New Jersey	3/20/20	na	1/10/22	9/24/21
New Mexico	4/4/19	3/18/20	na	na
New York	1/1/16	6/17/20	na	na
North Carolina	na	4/24/20	na	7/1/20
North Dakota	na	3/25/20	1/15/19	na
Ohio	na	3/20/20	7/1/21	4/27/21
Oklahoma	1/1/22	na	1/18/19	4/29/19
Oregon	6/1/21	4/6/20	na	na
Pennsylvania	na	4/20/20	10/26/16	5/8/20
Rhode Island	3/18/20	na	6/29/22	1/1/23
South Carolina	na	3/19/20	na	na
South Dakota	na	8/22/21	3/12/15	na
Tennessee	na	3/25/20	2/9/17	5/11/21
Texas	na	3/17/20	6/7/21	6/10/19
Utah	3/22/21	3/25/20	3/20/15	3/17/17
Vermont	3/16/20	na	12/21/17	na
Virginia	na	3/19/20	na	4/11/20
Washington	1/1/21	5/3/21	1/17/17	3/4/22
West Virginia	7/1/21	na	3/31/15	4/21/21
Wisconsin	na	3/1/20	12/14/15	2/4/22
Wyoming	na	na	2/27/15	na

[a] IMLC: Interstate Medical Licensure Compact. [b] PSYPACT: Psychology Interjurisdictional Compact. [c] CMHC: Community Mental Health Center.

eAppendix 2. Assessing Differential Time Trends in Telehealth Availability by Rurality and Race/Ethnicity

	Omnibus chi-square p-value
Interaction term	
(Rural) X (Year)	<0.001
(% Black Residents) x (Year)	0.05
(% Hispanic Residents) x (Year)	0.18

Note: Differential time trends were tested in a single regression model that included interaction terms for (Rural) X (Year), (% Black Residents) x (Year), and (% Hispanic Residents) x (Year). Year was treated as a categorical variable; % Black Residents and % Hispanic Residents were coded as four-level categorical variables, as in primary analyses. The regression model included state and year fixed effects and standard errors clustered at the facility level. Differential time trends were tested using an omnibus chi-square test for each interaction term. A statistically significant omnibus test indicated the presence of differential time trends across groups.

© 2023 McBain RK et al. *JAMA Network Open*

eAppendix 3. Assessing Differential Associations Between State Policy and Telehealth Availability by Rurality and Race/Ethnicity

	Omnibus chi-square p-value			
	Payment Parity	*Audio-Only Authorization*	*IMLC Compact*	*PSYPACT Compact*
Interaction term				
(% Black Residents) x (Policy)	<0.001	<0.001	0.31	0.91
(% Hispanic Residents) x (Policy)	0.15	0.01	0.07	0.09
(Rural) x (Policy)	0.02	<0.001	0.02	0.30

Note: For each policy, we estimated three separate regression models to assess (Rural) X (Policy), (% Black Residents) x (Policy), and (% Hispanic Residents) x (Policy), respectively. % Black Residents and % Hispanic Residents were coded as four-level categorical variables, as in primary analyses. Regression models included state and year fixed effects and standard errors clustered at the facility level. Heterogeneity regarding policy association was tested using an omnibus chi-square test for the given interaction term. A statistically significant omnibus test indicated the presence of differential policy associations across groups.

© 2023 McBain RK et al. *JAMA Network Open*

eAppendix 4. Predictive Margins Assessing Differential Associations Between State Policy and Telehealth Availability by Rurality and Race/Ethnicity

I. PAYMENT PARITY

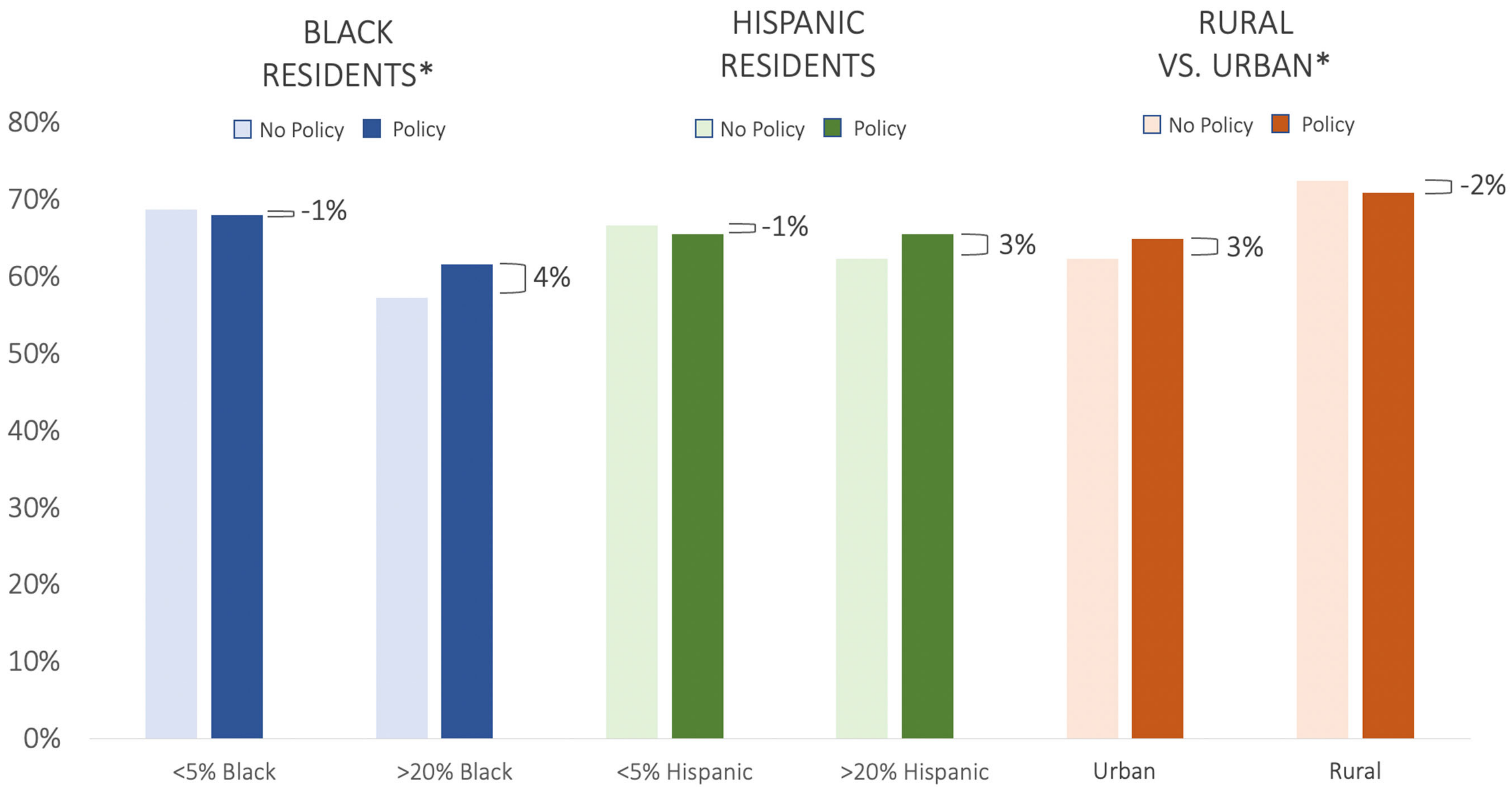

Note: Predictive margins were generated from regression models described in Appendix III. Asterisks (*) denote significant ($p<0.05$) differential effects according to omnibus chi-square tests: the relationship between policy implementation and telehealth availability was greater in some communities (e.g., communities that

© 2023 McBain RK et al. *JAMA Network Open*

were >20% Black) compared to others (e.g., communities that were <5% Black). Middle categories for % Black and % Hispanic were suppressed for parsimony in this presentation.

II. AUDIO-ONLY AUTHORIZATION

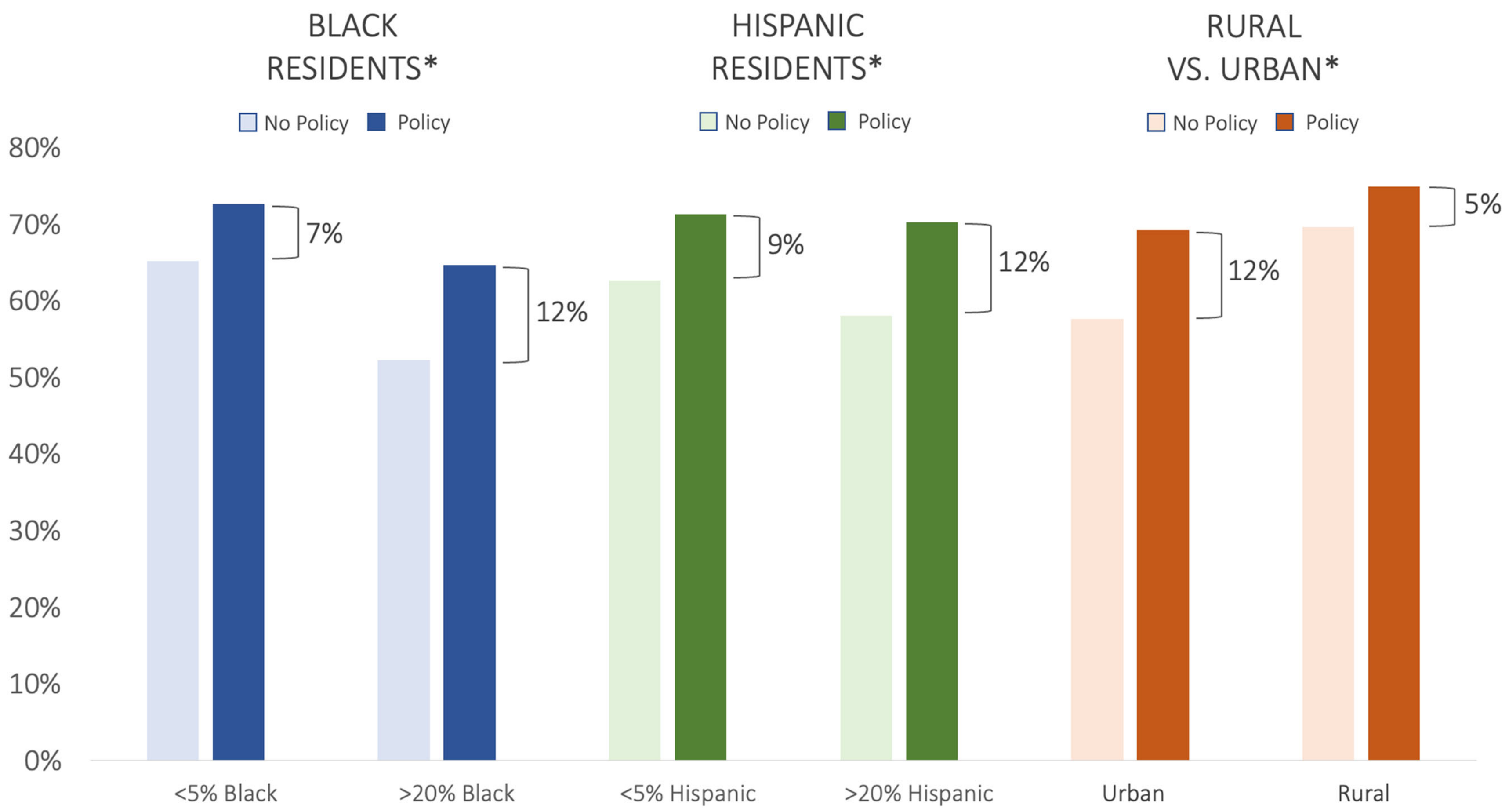

Note: Predictive margins were generated from regression models described in Appendix III. Asterisks (*) denote significant (p<0.05) differential effects according to omnibus chi-square tests: the relationship between policy implementation and telehealth availability was greater in some communities (e.g., communities that

© 2023 McBain RK et al. *JAMA Network Open*

were >20% Black) compared to others (e.g., communities that were <5% Black). Middle categories for % Black and % Hispanic were suppressed for parsimony in this presentation.

III. IMLC COMPACT

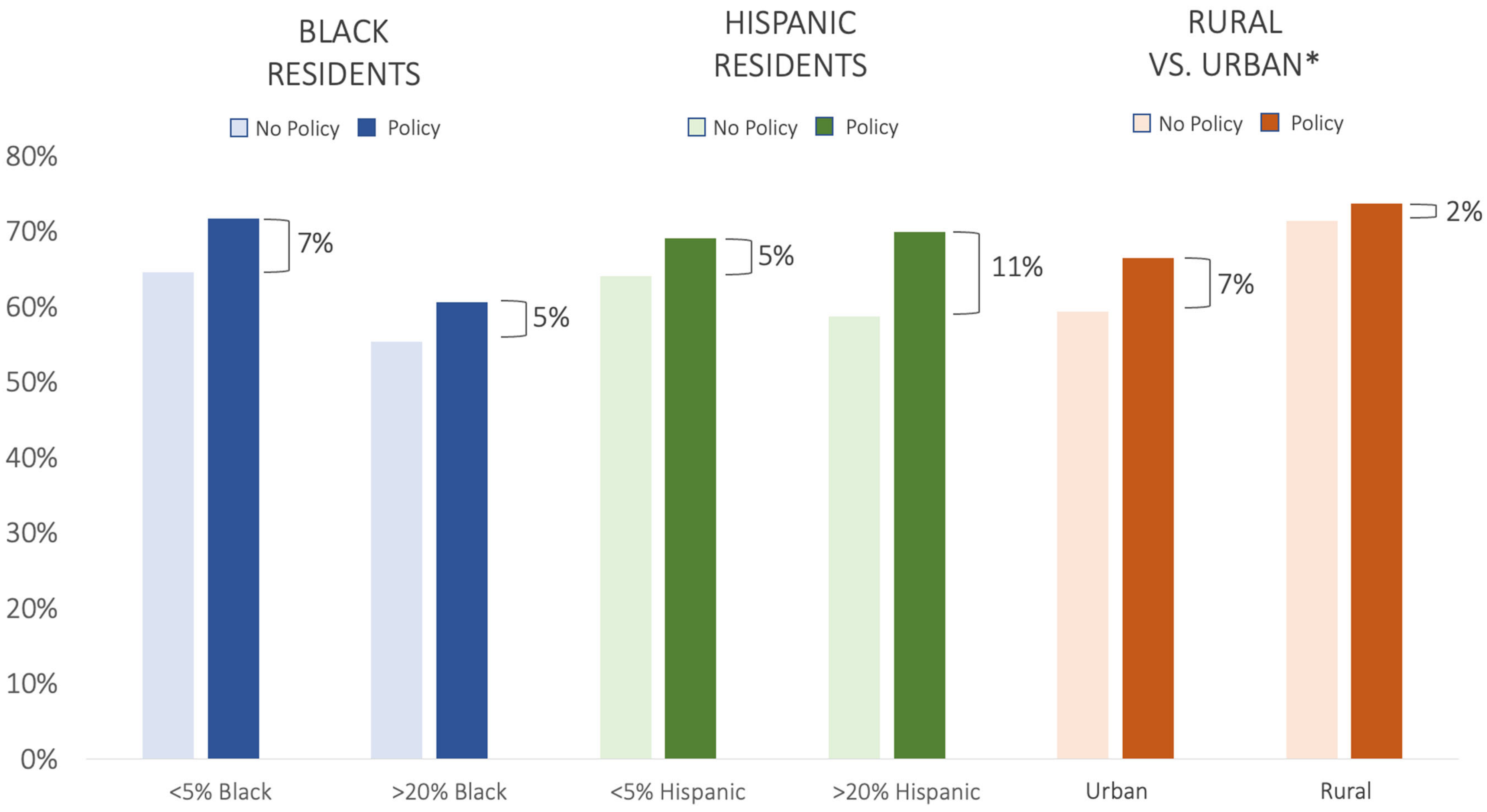

© 2023 McBain RK et al. *JAMA Network Open*

Note: Predictive margins were generated from regression models described in Appendix III. Asterisks (*) denote significant ($p<0.05$) differential effects according to omnibus chi-square tests: the relationship between policy implementation and telehealth availability was greater in some communities (e.g., communities that were urban) compared to others (e.g., communities that were rural). Middle categories for % Black and % Hispanic were suppressed for parsimony in this presentation.

IV. PSYPACT COMPACT

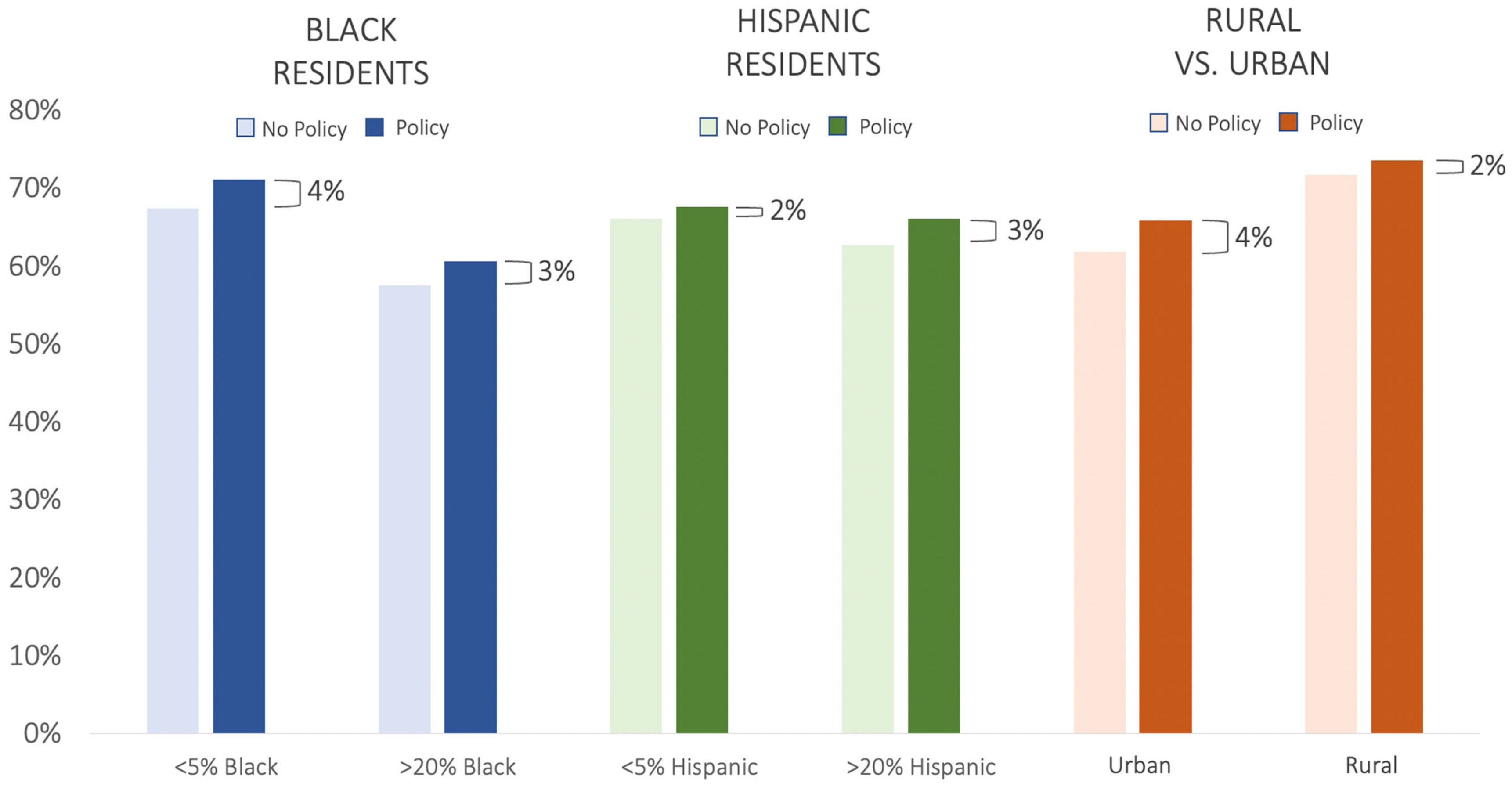

© 2023 McBain RK et al. *JAMA Network Open*

Note: Predictive margins were generated from regression models described in Appendix III. Asterisks (*) denote significant ($p<0.05$) differential effects according to omnibus chi-square tests: the relationship between policy implementation and telehealth availability was greater in some communities (e.g., communities that were >20% Black) compared to others (e.g., communities that were <5% Black). Middle categories for % Black and % Hispanic were suppressed for parsimony in this presentation.

© 2023 McBain RK et al. *JAMA Network Open*
