# Supplementary material for: Expansion of Telehealth Availability for Mental Health Care After State-Level Policy Changes From 2019 to 2022
Source: JAMA Netw Open. 2023 Jun 13;6(6):e2318045. doi: 10.1001/jamanetworkopen.2023.18045 (PMC10265313; doi:10.1001/jamanetworkopen.2023.18045)
Supplement: Supplement 2. — Data Sharing Statement [file jamanetwopen-e2318045-s002.pdf]

Data Sharing Statement

McBain. Expansion of Telehealth Availability for Mental Health Care After State-Level Policy Changes From 2019 to 2022. *JAMA Netw Open*. Published June 13, 2023.
doi:10.1001/jamanetworkopen.2023.18045

Data
Data available: No

Additional Information
Explanation for why data not available: Data will be made available upon request from the corresponding author.
